# Supplementary material for: Prediction of Hemorrhagic Complication after Thrombolytic Therapy Based on Multimodal Data from Multiple Centers: An Approach to Machine Learning and System Implementation
Source: J Pers Med. 2022 Dec 12;12(12):2052. doi: 10.3390/jpm12122052 (PMC9782609; doi:10.3390/jpm12122052)
Supplement: Supplementary file 1 [file jpm-12-02052-s001.zip › Supplementary_Materials/Supplementary_Materials.pdf]

Journal of Personalized Medicine

Supplementary materials

Table S1. Baseline analysis of training set, internal verification set and external verification set.

**Variables**	**train short (332)**			**internal validation (83)**			**external validation (102)**			**p**
	N (179)	P (153)	p	N (46)	P (37)	p	N (57)	P (45)	p	
**Demographic**										
gender, n (%)			0.745			0.055			0.657	0.059
Male	113 ±63.1	93 ±60.8		33 ±71.7	18 ±48.6		41 ±71.9	35 ±77.8		
Female	66 ±36.9	60 ±39.2		13 ±28.3	19 ±51.4		16 ±28.1	10 ±22.2		
Age, years, mean±SD	66.35±12.94	68.60 ±11.76	0.101	64.13±10.38	68.73 ±16.43	0.124	68.11 ±11.73	64.51 ±13.84	0.159	0.67
Smoking, mean±SD	301.17 ±507.39	267.19 ±478.24	0.533	390.22 ±538.06	304.59 ±858.46	0.581	291.23 ±412.43	405.11 ±1317.97	0.539	0.577
Drinking, mean±SD	1461.45 ±3492.40	1351.31 ±3503.83	0.775	1202.72 ±2380.20	781.08 ±1963.39	0.389	1538.60 ±3651.90	366.67 ±802.84	0.037	0.409
**Past medical history**										
History of stroke, n (%)			0.001			0.009			0.047	0.055
No-history-of-stroke	156(87.2)	109 (71.2)		40 (87.0)	22 (59.5)		54 (94.7)	36 (80.0)		
Have-a-history-of-stroke	23 (12.8)	44 (28.8)		6 (13.0)	15 (40.5)		3 (5.3)	9 (20.0)		
Diabetes, n (%)			0.011			0.843			0.967	0.234
No-diabetes	138 (77.1)	97 (63.8)		29 (63.0)	25 (67.6)		43 (75.4)	35 (77.8)		
Diabetes-mellitus	41 (22.9)	55(36.2)		17 (37.0)	12 (32.4)		14 (24.6)	10 (22.2)		
Atrial fibrillation, n (%)			<0.001			<0.001			0.172	0.03
No-atrial-fibrillation	156 (87.2)	97 (63.4)		44 (95.7)	23 (62.2)		53 (93.0)	37 (82.2)		
Atrial-fibrillation	23 (12.8)	56(36.6)		2 (4.3)	14 (37.8)		4(7.0)	8 (17.8)		
**Clinical manifestation**										
Onset time, hours, mean±SD	5.95 ±2.78	4.78 ±2.72	<0.001	6.12 ±2.84	4.58 ±1.87	0.006	6.41 ±2.95	4.34 ±2.14	<0.001	0.959
SBP, mm Hg, mean±SD	153.11 ±24.63	149.41 ±26.12	0.184	156.15 ±21.87	147.24 ±29.77	0.120	156.00 ±26.02	151.11 ±20.48	0.304	<0.001
DBP, mm Hg, mean±SD	86.66±13.87	87.18 ±15.64	0.749	89.00 ±14.22	85.14 ±13.43	0.211	88.79 ±13.36	86.93 ±14.02	0.497	<0.001
NIHSS OA, mean±SD	6.52±5.51	13.06 ±10.21	<0.001	4.87 ±2.93	13.11±6.79	<0.001	4.35 ±2.76	15.27 ±6.08	<0.001	<0.001
TOAST CF, n (%)			<0.001			0.001			0.002	0.007
Type I: Large athero-sclerotic type	119(66.5)	72 (47.1)		33(71.7)	13 (35.1)		33 (57.9)	30 (66.7)		

Type II: cardioembolic stroke	33 (18.4)	71 (46.4)		9 (19.6)	20 (54.1)		5 (8.8)	13 (28.9)		
Type III: arteriole type	22 (12.3)	4 (2.6)		4 (8.7)	1 (2.7)		16 (28.1)	1(2.2)		
Type IV: other reasons	1 (0.6)	1 (0.7)		0 (0.0)	3 (8.1)		2(3.5)	1 (2.2)		
Type V: Unknown cause	4(2.2)	5 (3.3)		4 (2.2)	5 (3.3)		1 (1.8)	0 (0.0)		
**Laboratory examination**										
Hemoglobin, g/L, mean±SD	73.15 ±64.56	85.49 ±61.14	0.076	74.08 ±69.14	77.25 ±62.35	0.829	7.44 ±1.63	10.59 ±3.80	<0.001	<0.001
Platelets, 10^9/L, mean±SD	177.18 ±101.76	179.47 ±85.97	0.827	163.11 ±57.86	171.19 ±95.46	0.635	136.33 ±15.99	129.16 ±17.73	0.034	<0.001
WBC, 10^9/L, mean±SD	103.23 ±107.98	91.57 ±117.46	0.347	109.25 ±111.86	91.21 ±103.93	0.453	229.30 ±96.21	200.73 ±85.55	0.121	<0.001
Neutrophils, 10^9/L, mean±SD	5.49 ±2.38	7.49 ±5.33	<0.001	5.38 ±2.49	7.86 ±3.69	<0.001	5.31 ±1.47	8.34 ±3.42	<0.001	0.862
Lymphocytes, 10^9/L, mean±SD	1.60 ±0.85	1.54 ±1.78	0.653	1.65 ±0.61	1.28 ±0.67	0.012	1.42 ±0.49	1.52 ±0.69	0.39	0.633
Monocytes, 10^9/L, mean±SD	0.48 ±0.21	0.64 ±0.27	<0.001	0.48 ±0.18	1.00 ±1.77	0.051	0.51 ±0.19	0.74 ±0.26	<0.001	0.05
TRONTL, mean±SD	4.31 ±3.11	7.49 ±8.44	<0.001	4.08 ±3.05	8.57 ±8.12	0.001	4.33 ±2.69	7.33 ±7.57	0.006	0.891
LTMR, mean±SD	3.86 ±3.13	2.84 ±3.55	0.006	3.82 ±1.87	2.76 ±3.76	0.101	3.25 ±2.00	2.35 ±1.38	0.011	0.294
AVOE, 10^9/L, mean±SD	0.13 ±0.18	0.17 ±0.36	0.176	0.13 ±0.13	0.18 ±0.33	0.362	0.15 ±0.17	0.04 ±0.04	<0.001	0.187
Total cho, mmol/L, mean±SD	4.40 ±1.02	4.63 ±1.42	0.092	4.40 ±1.07	4.66 ±1.16	0.287	4.40 ±0.88	5.24 ±1.35	<0.001	0.133
Triglycerides, mmol/L, mean±SD	1.61 ±1.09	1.67 ±1.47	0.664	1.75 ±1.08	1.47 ±0.74	0.178	1.56 ±1.51	1.95 ±1.61	0.215	0.78
HDL, mmol/L, mean±SD	1.13 ±0.38	1.29 ±0.46	<0.001	1.12 ±0.29	1.39 ±0.57	0.006	1.20 ±0.37	1.46 ±0.73	0.02	0.115
LDL, mmol/L, mean±SD	2.81 ±0.90	2.70 ±1.02	0.326	2.71 ±1.03	2.82 ±1.19	0.636	2.78 ±0.77	2.98 ±1.09	0.281	0.603
BUN, mmol/L, mean±SD	6.11 ±3.71	6.87 ±3.42	0.056	5.68 ±2.19	7.08 ±4.32	0.059	6.02 ±2.72	6.05 ±3.10	0.959	0.544
Creatinine, mmol/L, mean±SD	78.15 ±52.94	81.12 ±45.55	0.587	78.72 ±28.96	86.98 ±64.06	0.437	79.25 ±58.50	88.67 ±35.90	0.345	0.743
Fast blood sugar, mean±SD	7.00 ±2.89	8.80 ±4.06	<0.001	7.56 ±2.80	8.66 ±3.47	0.112	7.06 ±2.51	8.95 ±3.65	0.003	0.87
GH, %, mean±SD	6.83 ±2.02	9.68 ±10.14	<0.001	7.23 ±1.86	7.69 ±2.20	0.303	6.73 ±1.76	9.08 ±8.06	0.035	0.623

INR, mean±SD	1.00 ±0.14	1.06 ±0.16	0.001	0.99 ±0.07	1.02 ±0.16	0.225	0.97 ±0.15	1.06 ±0.14	0.002	0.311
PFDP, μg/mL, mean±SD	3.88 ±6.55	9.51 ±14.59	<0.001	2.73 ±4.60	14.62 ±21.76	0.001	3.73 ±11.67	9.36 ±18.17	0.061	0.573
D dimer, mg/L, mean±SD	1.46 ±2.72	3.34 ±5.48	<0.001	1.00 ±2.17	6.11 ±10.27	0.002	1.55 ±4.40	3.92 ±8.23	0.065	0.352
BNPR, ng/L, mean±SD	684.37 ±1372.73	1576.93 ±2633.39	<0.001	547.07 ±1696.74	1580.38 ±2103.96	0.015	398.09 ±1242.34	1776.09 ±2215.27	<0.001	0.892

Categorical variables are represented by the number (percent), and continuous variables are represented by mean (± standard deviation); SBP, systolic blood pressure; DBP, diastolic blood pressure; NIHSS OA, NIHSS on admission; TOAST CF, TOAST classification; WBC, white blood cells; TRONTL, the ratio of neutrophils to lymphocytes; LTMR, lymphocyte to monocyte ratio; AVOE, absolute value of eosinophils ; Total cho, total cholesterol; GH, glycated hemoglobin ; INR, international normalized ratio; PFDP, plasma fibrin degradation products; BNPP, BNP precursor.
Note: The description of smoking and drinking habits in the table uses the Smoking index and Drinking index. Smoking index= the number of cigarettes per day * Years of smoking; Drinking index= Daily alcohol consumption (g) * Years of drinking (45~55 degree Chinese liquor).

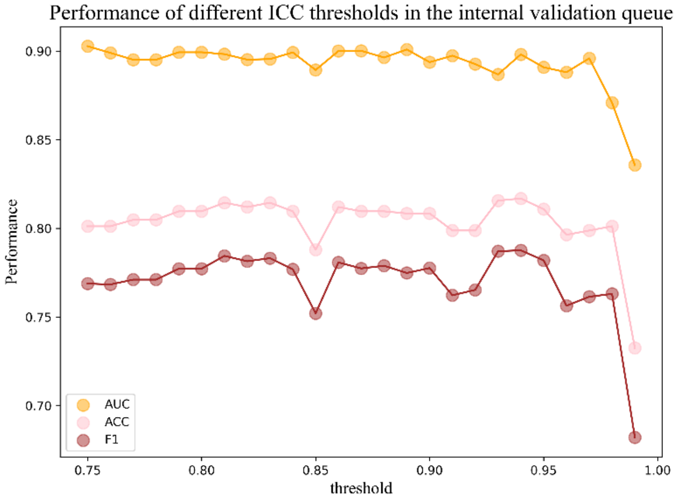

Figure S1. ICC analysis for feature screening threshold analysis.

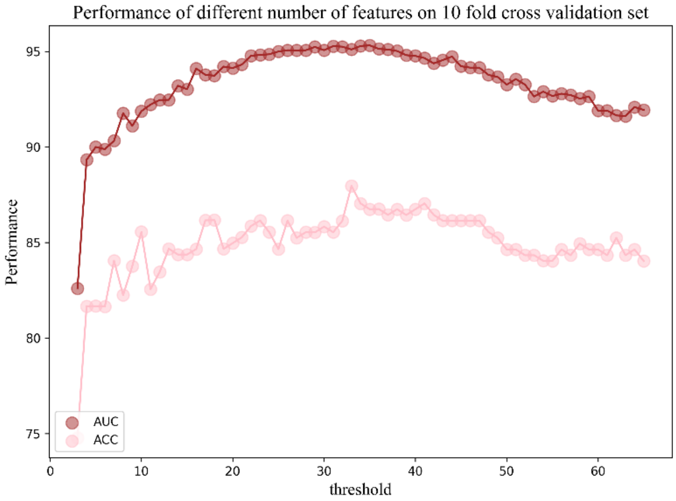

(a) LR (best threshold=33)

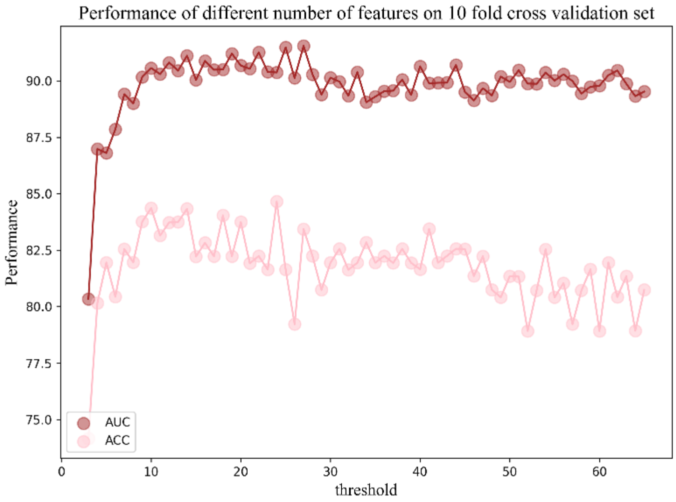

(b) RF (best threshold=24)

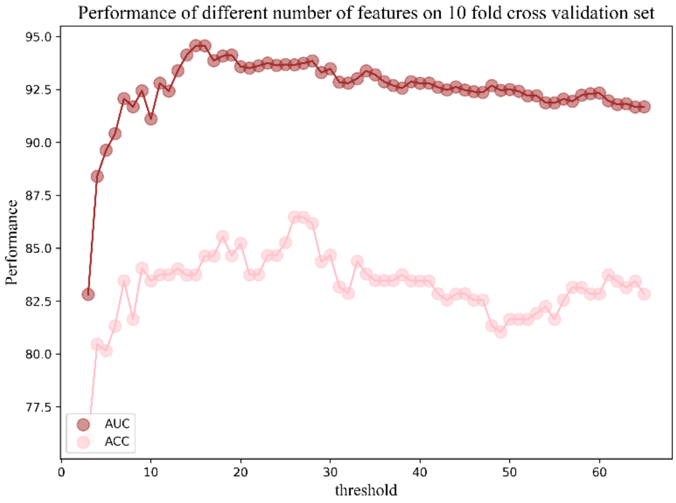

(c) SVM (best threshold=26)

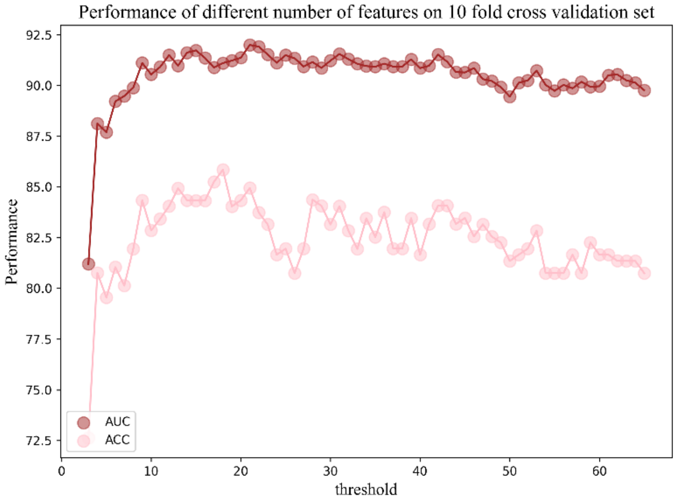

(d) XGB (best threshold=18)

Figure S2. Performance of each model when selecting different number of features, figures a, b, c and d are the analysis of the number of selected features of LR, RF, SVM and XGBoost respectively.

Table S2. Results of 10 - fold cross validation of each model on the training set.

Classifier	LR				RF				SVM				XGB			
	T cohort	V cohort	IV cohort	EV cohort	T cohort	V cohort	IV cohort	EV cohort	T cohort	V cohort	IV cohort	EV cohort	T cohort	V cohort	IV cohort	EV cohort
**ACC**	0.9033	0.8795	0.8675	0.8265	0.9210	0.8135	0.8289	0.8020	0.9163	0.8793	0.8578	0.8147	0.9592	0.8585	0.8554	0.8078
**AUC**	0.9695	0.9484	0.8923	0.9059	0.9822	0.8950	0.9109	0.8922	0.9661	0.9483	0.8882	0.8929	0.9927	0.9263	0.9332	0.9044
**SEN**	0.9187	0.8892	0.7784	0.8000	0.9426	0.8367	0.7676	0.7644	0.9288	0.9012	0.7486	0.7867	0.9695	0.8700	0.7622	0.7778
**SPE**	0.8901	0.8699	0.9391	0.8474	0.9025	0.7918	0.8789	0.8316	0.9056	0.8585	0.9457	0.8368	0.9504	0.8477	0.9304	0.8316
**PPV**	0.8774	0.8684	0.9117	0.8055	0.8926	0.7820	0.8358	0.7818	0.8939	0.8570	0.9183	0.7921	0.9437	0.8443	0.9011	0.7851
**NPV**	0.9276	0.9124	0.8406	0.8429	0.9486	0.8627	0.8253	0.8179	0.9371	0.9180	0.8241	0.8326	0.9734	0.8881	0.8309	0.8273
**F1**	0.8975	0.8721	0.8396	0.8027	0.9168	0.8040	0.7996	0.7727	0.9110	0.8747	0.8244	0.7892	0.9563	0.8519	0.8239	0.7805

T cohort, Training cohort; V cohort, Validation cohort; IV cohort, Internal Validation cohort; EV cohort, external Validation cohort.

Table S3. Delong test results of internal validation and external validation of each model.

DelongTest (internal validation set)								
**Classifier**	LR		RF		SVM		XGB	
	z	p	z	p	z	p	z	p
LR	0.0000	1.0000	0.5840	0.5592	-0.2086	0.8348	1.2775	0.2014
RF	-0.5840	0.5592	0.0000	1.0000	-0.6091	0.5425	1.2125	0.2253
SVM	0.2086	0.8348	0.6091	0.5425	0.0000	1.0000	1.3050	0.1919
XGB	-1.2775	0.2014	-1.2125	0.2253	-1.3050	0.1919	0.0000	1.0000
DelongTest (external validation set)								
**Classifier**	LR		RF		SVM		XGB	
	z	p	z	p	z	p	z	p
LR	0.0000	1.0000	-0.4197	0.6747	-1.4848	0.1376	0.2246	0.8223
RF	0.4197	0.6747	0.0000	1.0000	-0.0333	0.9734	0.9029	0.3666
SVM	1.4848	0.1376	0.0333	0.9734	0.0000	1.0000	0.6864	0.4925
XGB	-0.2246	0.8223	-0.9029	0.3666	-0.6864	0.4925	0.0000	1.0000

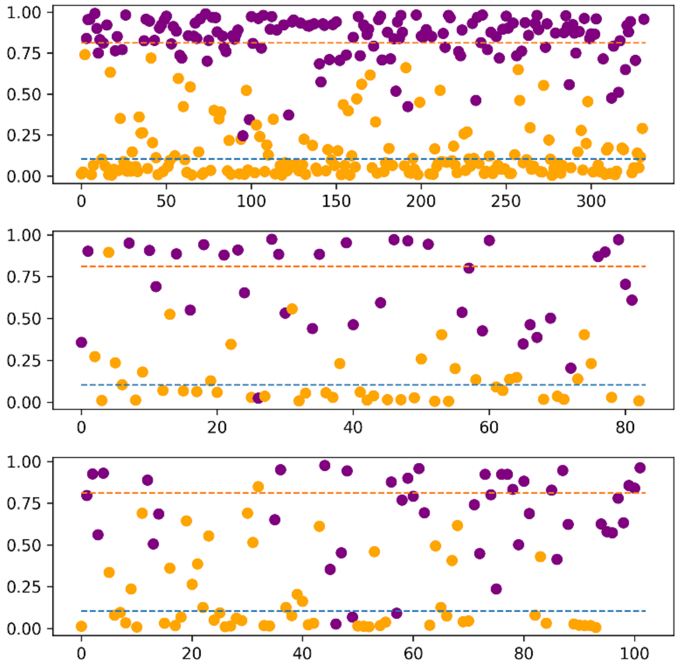

Figure S3. Scatter diagram of model prediction results, from top to bottom, is the training set, internal verification set, and external verification set.
