# Supplementary figures and images for: Prediction of Hemorrhagic Complication after Thrombolytic Therapy Based on Multimodal Data from Multiple Centers: An Approach to Machine Learning and System Implementation
Source: J Pers Med. 2022 Dec 12;12(12):2052. doi: 10.3390/jpm12122052 (PMC9782609; doi:10.3390/jpm12122052)

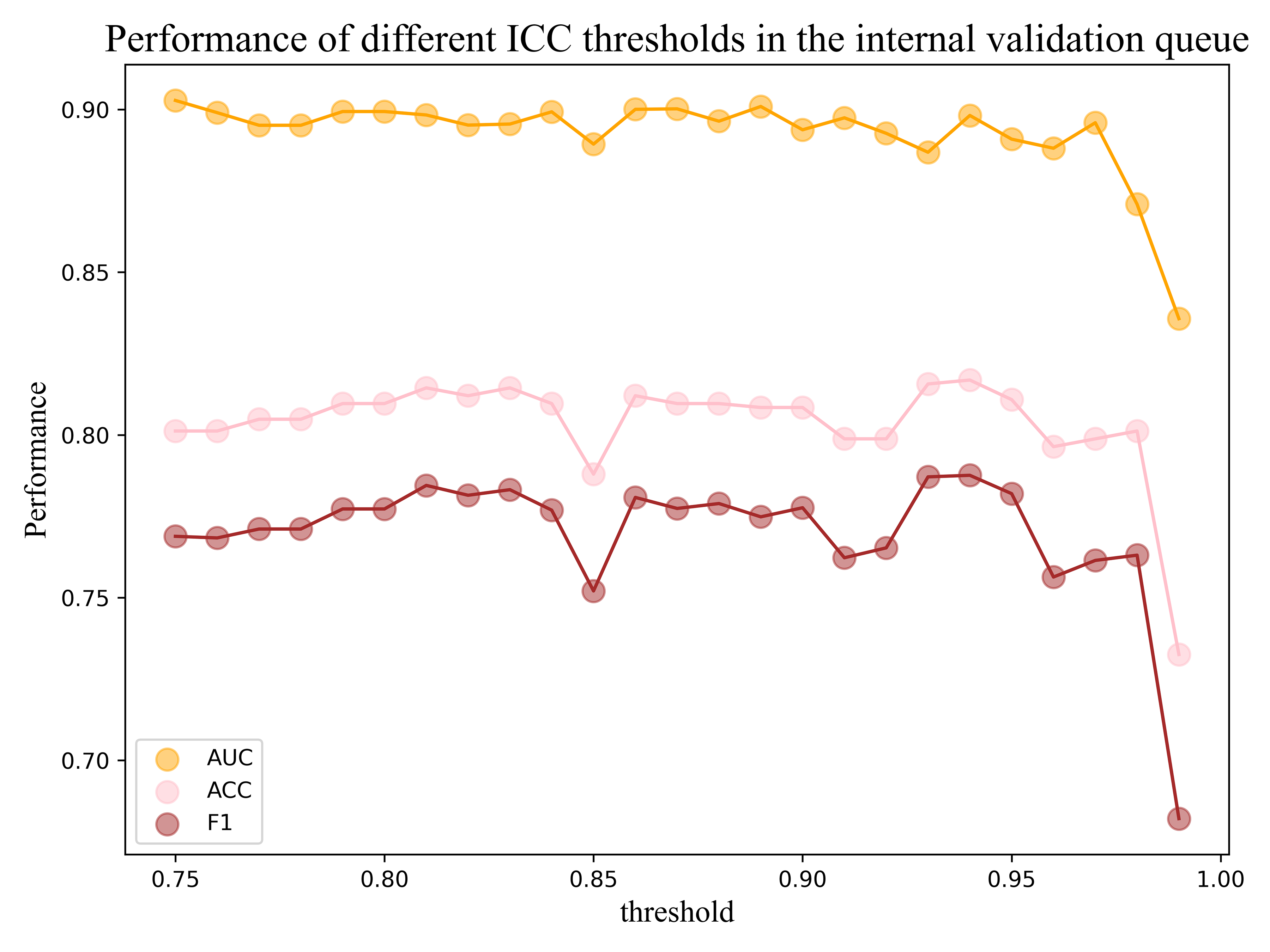

Supplement: Supplementary file 1 [file jpm-12-02052-s001.zip › Supplementary_Materials/figure_S1.jpg]

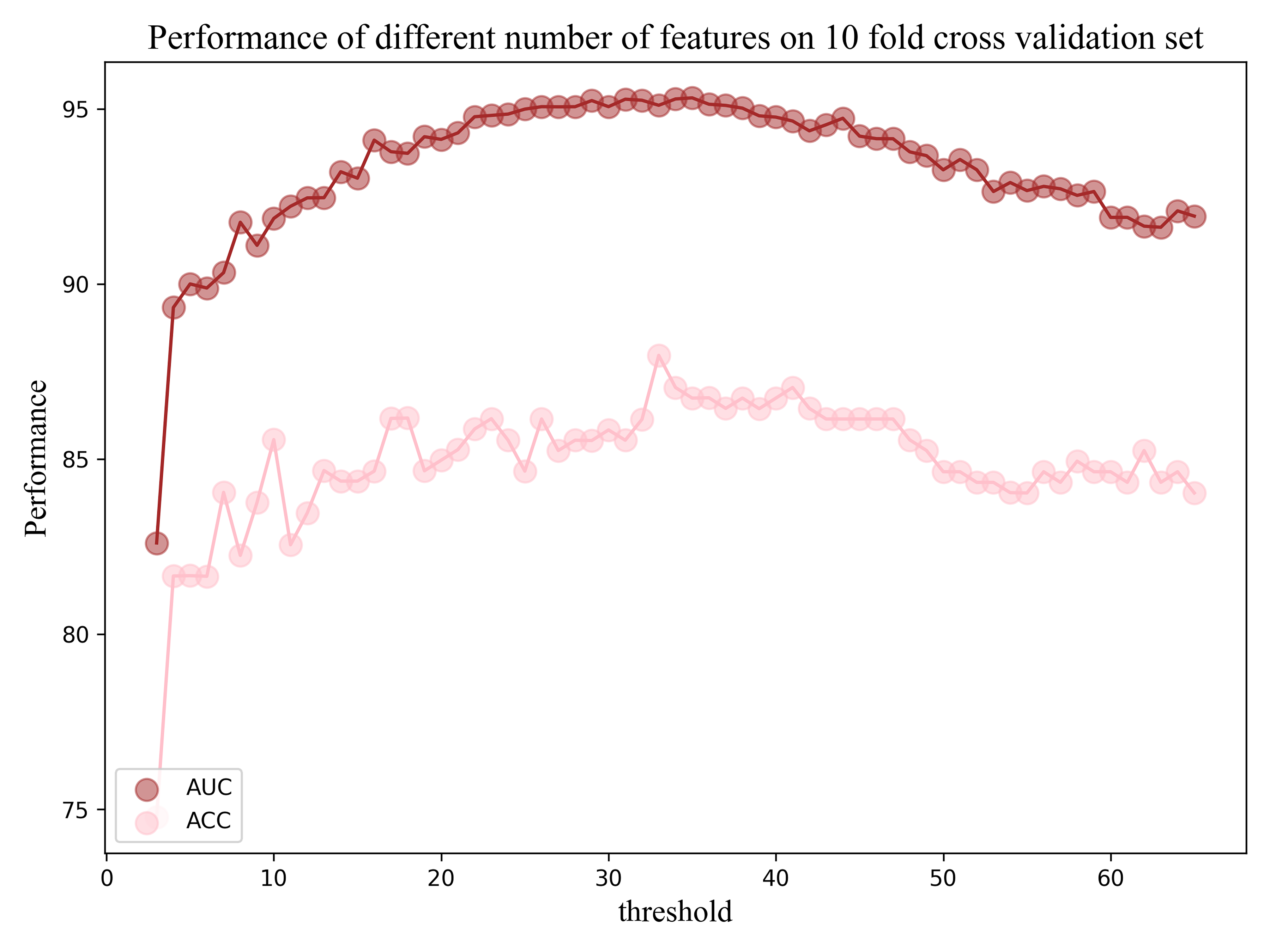

Supplement: Supplementary file 1 [file jpm-12-02052-s001.zip › Supplementary_Materials/figure_S2(a).jpg]

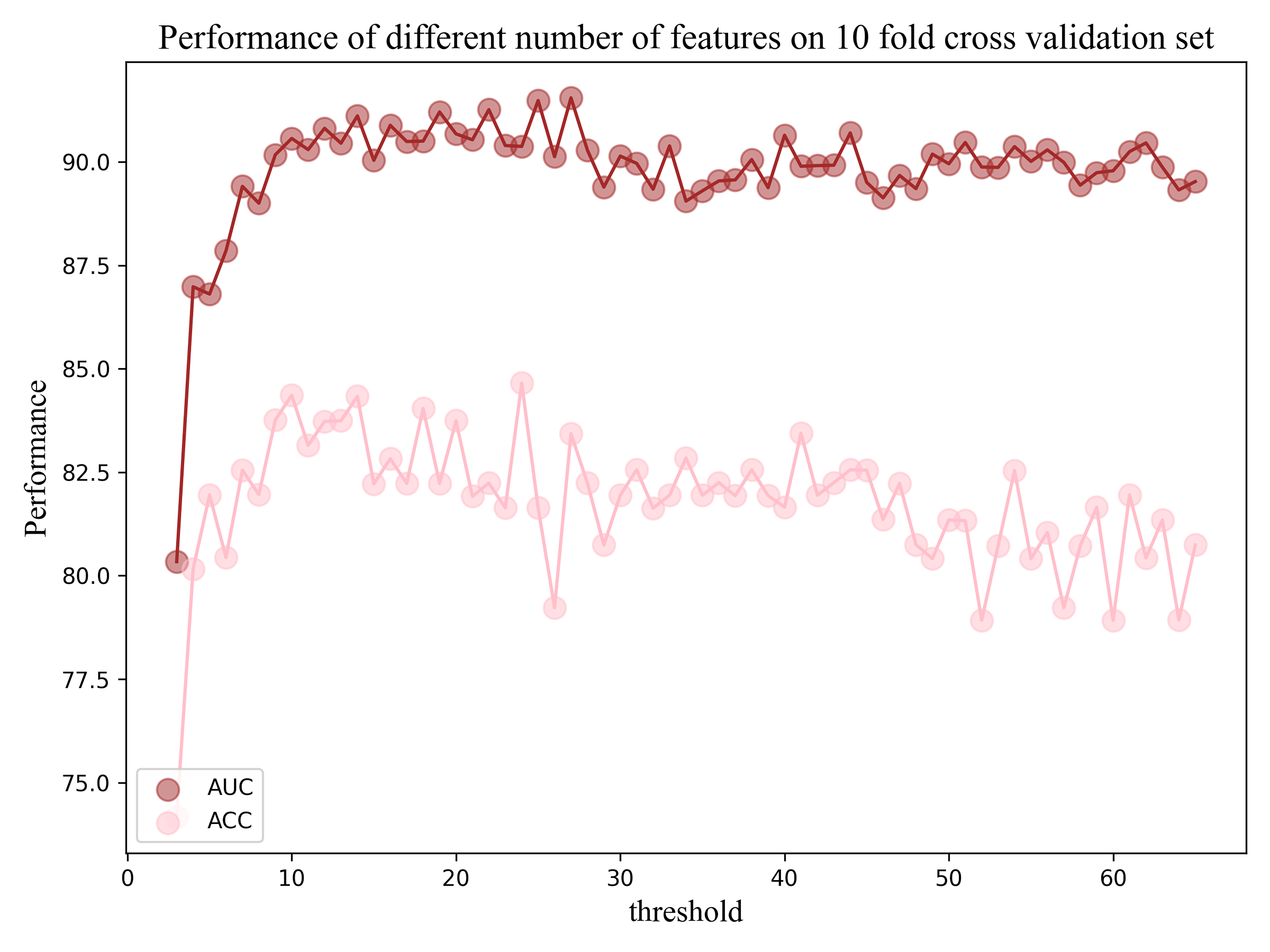

Supplement: Supplementary file 1 [file jpm-12-02052-s001.zip › Supplementary_Materials/figure_S2(b).jpg]

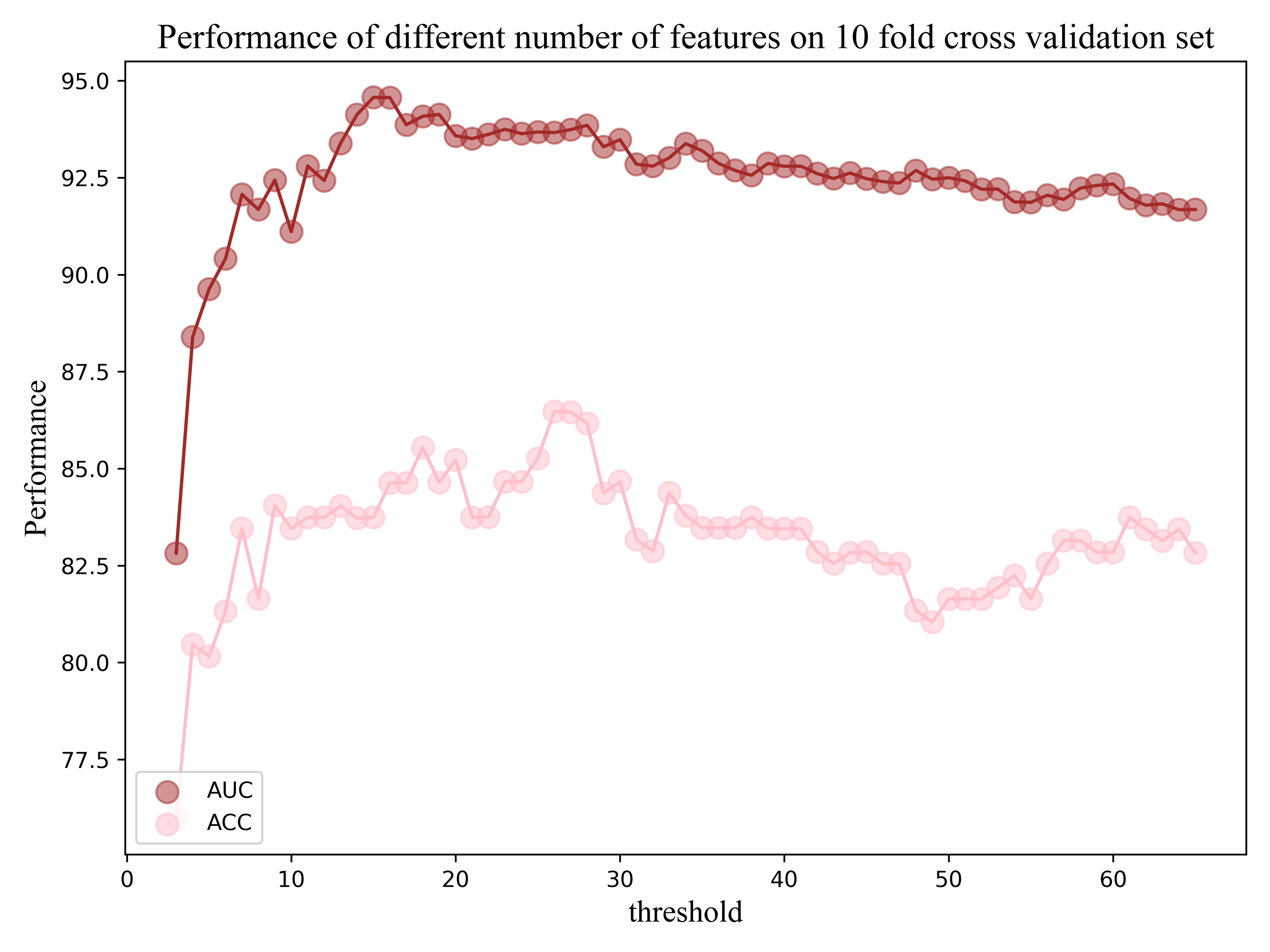

Supplement: Supplementary file 1 [file jpm-12-02052-s001.zip › Supplementary_Materials/figure_S2(c).jpg]

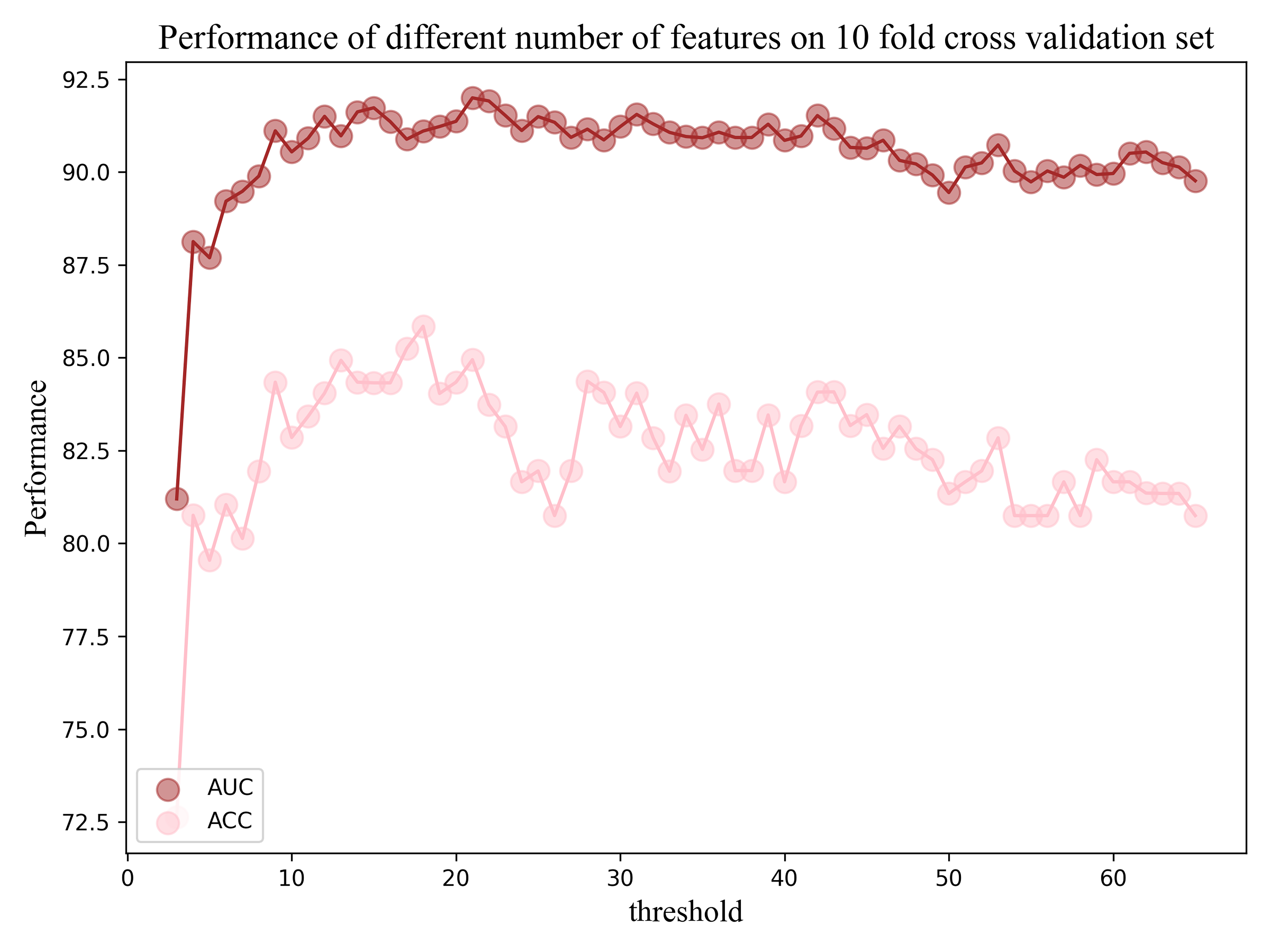

Supplement: Supplementary file 1 [file jpm-12-02052-s001.zip › Supplementary_Materials/figure_S2(d).jpg]

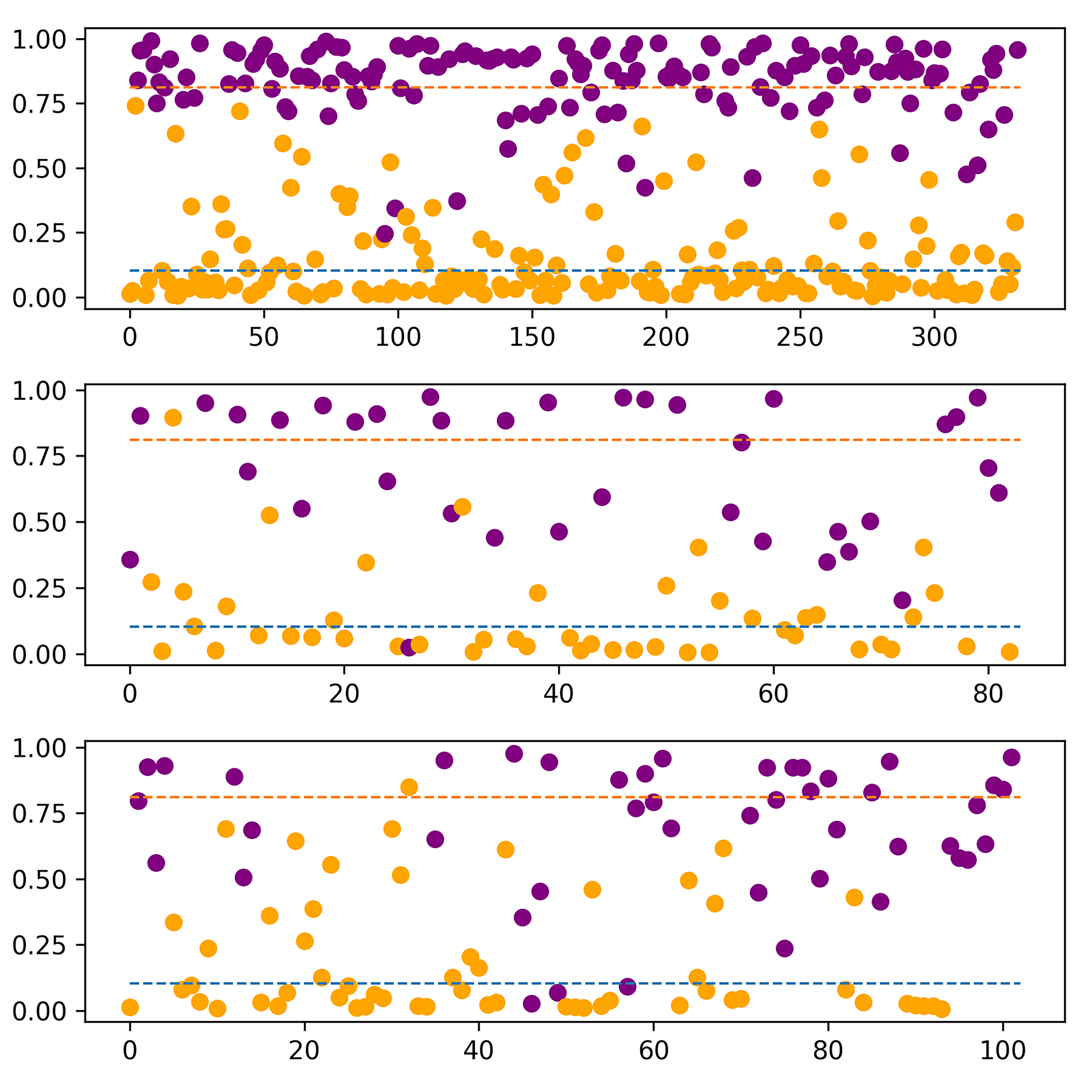

Supplement: Supplementary file 1 [file jpm-12-02052-s001.zip › Supplementary_Materials/figure_S3.jpg]
